# Infant Safe Sleep Promotion: Increasing Capacity of Child Protective Services Employees

**DOI:** 10.3390/ijerph18084227

**Published:** 2021-04-16

**Authors:** Carolyn R. Ahlers-Schmidt, Christy Schunn, Ashley M. Hervey, Maria Torres, Cherie Sage, Martha Henao, Stephanie Kuhlmann

**Affiliations:** 1 Center for Research for Infant Birth and Survival (CRIBS), Department of Pediatrics, School of Medicine-Wichita, University of Kansas, Wichita, KS 67208, USA; ahervey@kumc.edu (A.M.H.); mhenaovilla@kumc.edu (M.H.); skuhlmann@kumc.edu (S.K.); 2 Kansas Infant Death and SIDS (KIDS) Network, Wichita, KS 67202, USA; edirector@kidsks.org (C.S.); ssidirector@kidsks.org (M.T.); 3 Safe Kids Kansas, Topeka, KS 66612, USA; Cherie.Sage@ks.gov

**Keywords:** sudden infant death syndrome, sleep-related infant death, child protective services

## Abstract

Sleep-related infant deaths, including Sudden Infant Death Syndrome (SIDS), are the number one cause of death in infants between 28 days and one year of life. Nearly half of families experiencing a sleep-related infant death in Kansas were involved with the Department of Children and Families Child Protective Services (CPS), making CPS staff a priority for safe sleep training. This study assessed the impact of the two-day Kansas Infant Death and SIDS (KIDS) Network Safe Sleep Instructor (SSI) train-the-trainer program on CPS staffs’ knowledge of the American Academy of Pediatrics safe sleep recommendations. Training was attended by 43 participants, 27 (63%) of whom were employed by CPS. CPS staff had significantly lower baseline knowledge on the 10-item pretest (*t =* 3.33, *p* = 0.002), but both CPS and other attendees showed significant improvement by posttest (*t =* 8.53, *p* < 0.001 and *t =* 4.44, *p* < 0.001, respectively). Following SSI certification, CPS SSIs provided more safe sleep training to professionals than other SSIs (1051 vs. 165, respectively), and both groups of SSIs were able to significantly increase the knowledge of their trainees. Overall, the KIDS Network SSI training was successful. The innovative partnership with CPS allowed for provision of training to a group not historically targeted for safe sleep education.

## 1. Introduction

The highest proportion of child maltreatment deaths in the United States (US) occur in infants less than 1 year of age [1]. Case management and family support services, such as those provided by child protective service (CPS) staff, are intended to reduce child abuse and neglect, and ultimately the fatalities resulting from these.

Sleep-related infant deaths, including Sudden Infant Death Syndrome (SIDS), are the number one cause of death in infants between 28 days and one year of life. Recommendations to reduce the risk of sleep-related infant deaths have been provided by the American Academy of Pediatrics (AAP) since the early 1990s and include having the infant sleep alone on their back in a clutter-free crib during naptime and nighttime [2]. However, many families fail to follow these recommendations for reasons including but not limited to a lack of knowledge, a lack of credible information sources, a lack of understanding of the mechanism of risk reduction, confusion from receiving inconsistent advice, parent preference or experience, and disruptions to their routine [3,4,5].

Effective safe sleep interventions from the US and other countries have focused on health messaging, education of professionals, breaking down barriers, utilizing culture and tradition, and addressing legislation and regulations [6]. Most professional education has prioritized health care or childcare professionals. In terms of outcomes, a systematic review of safe sleep interventions found that the majority of studies reported significant improvements in the intention or use of supine positioning [5]. For example, an education intervention with Women, Infant, and Children (WIC) participants found significant increases at 6-month follow-up regarding self-report of back position and not bed-sharing [6]. In contrast, outcomes regarding sleep location/surface, items in the crib, and sharing safe sleep information with others varied between studies [5].

Factors such as low income/employment, housing instability, and parent mental health have also been identified as risk factors for both child abuse and maltreatment [7], and sleep-related infant deaths [2,8,9,10]. However, the literature regarding the interaction of CPS and sleep-related deaths is exceedingly sparse [11] and tends to focus on the prevalence of CPS involvement with families who experience a SIDS death. For example, in San Diego County, California, out of nearly 400 cases of SIDS, 11% had a CPS referral for the infant who died and 29% had a referral within the previous five years [11]. Referrals were mostly due to neglect and were substantiated in 38% and 27% of cases, respectively. No identified studies examined the education of or interventions by CPS to specifically reduce such sleep-related deaths.

In 2019, the Kansas State Child Death Review Board reported that 49% of families experiencing a sleep-related infant death had current or past Kansas Department of Children and Families (DCF) CPS involvement [12]. On 1 July 2019, after a discussion with the Kansas Infant Death and SIDS (KIDS) Network and SAFE Kids Kansas, DCF instituted a policy mandating that CPS staff assess sleep environments for all infants under 1 year of age [13]. To ensure accurate knowledge of the safe sleep recommendations [2], DCF also encouraged and subsidized staff attendance at the KIDS Network Annual Safe Sleep Instructor (SSI) training.

The purpose of this project was to assess CPS staff’s knowledge of the American Academy of Pediatrics (AAP) safe sleep recommendations [2] after attending the SSI training, to compare their knowledge to SSI trainees from other organizations (other SSI trainees), and to evaluate their ability to disseminate safe sleep knowledge to caregivers and professionals following certification.

## 2. Materials and Methods

### 2.1. Fall 2019 SSI Certification Training

The KIDS Network hosts an annual Safe Sleep Instructor (SSI) training to certify professionals as safe sleep educators [14,15]. Previous attendees included physicians, nurses, coordinators for maternal/child health programs (e.g., WIC), prenatal/infant care educators, and childcare license surveyors; no CPS staff previously attended.

The curriculum is based on the AAP safe sleep recommendations [2] and is updated each year based on current research and guidelines. Topics include diagnosis of sleep-related deaths, including SIDS; data trends and disparities in sleep-related infant deaths; safe sleep locations (e.g., parent’s room), surfaces (e.g., crib), items in the sleep environment (e.g., fitted sheet), and positions (e.g., supine); risks of tobacco use; protective quality of breastfeeding; and recommended practices related to temperature regulation and pacifier use. Attendees learn to provide 1 h of safe sleep training covering the topics listed above, including strategies to communicate safe sleep messages to both professionals and infant caregivers. They also learn to facilitate a safe sleep crib demonstration and how to host a Safe Sleep Community Baby Shower [15,16] or Crib Clinic. Instruction is also provided on how to be an effective presenter, on how to tailor education to the most disparate populations, on how to identify and engage key community stakeholders, on data collection and data entry, and on grant writing. Education formats include didactics, demonstration, hands-on activities, small group discussions, videos, and panel discussions. Following training, SSIs are certified to educate parents/caregivers, childcare providers, health care providers, and other members of their communities about safe sleep practices. SSIs are tasked with providing safe sleep training to at least 10 professionals and with hosting one Safe Sleep Community Baby Shower or Crib Clinic within 9 months of certification.

CPS staff and other professionals attended the two-day SSI training in fall 2019. All participants completed a 10-item pre- and post-training knowledge assessment at the beginning of the training and immediately following the training, respectively. The multiple choice questions addressed the definition of SIDS; research regarding the most effective ways to share safe sleep information with families; and the specific AAP recommendations regarding sleep location, sleep surface, sleep position, items in the sleep environment, increased risk due to tobacco use, decreased risk due to breastfeeding and pacifier use, temperature regulation, and multiple infants (e.g., twins). The posttest also included self-assessments of knowledge change and levels of agreement with the AAP safe sleep recommendations [2]. In addition, all participants were evaluated on their ability to conduct a safe sleep crib demonstration based on a priori criteria. Pre- and posttest data were deidentified and entered into a secure database by KIDS Network staff. Finally, participants provided feedback on their satisfaction with the training.

### 2.2. Post-Certification Dissemination of Safe Sleep Information by SSIs

For training conducted by certified SSIs, once they returned to their communities, pre- and posttest data were collected, deidentified, and entered into a secure database. Parent and caregiver training used a 4-item, multiple choice survey that assessed (1) sleep position, (2) location and surface, (3) items in sleep environment, and (4) discussion with other infant caregivers who might put the infant to sleep. Professional training utilized the same 10-item, multiple choice test as the SSI training. Participants were encouraged to complete the pre- and posttests in their entirety. However, due to the voluntary nature, respondents had the right to skip questions. Missing data on knowledge questions were coded as incorrect responses. All reported data were collected between 1 October 2019 and 13 July 2020. Due to restrictions on gathering size and in-person meetings related to the COVID-19 pandemic, Safe Sleep Community Baby Showers and Crib Clinics were not conducted during this time.

Descriptive statistics were summarized using means (standard deviations) and frequencies (percentages). Differences within groups were evaluated by paired samples t-test. Comparisons between CPS versus other SSIs in terms of SSI training knowledge scores, self-assessed knowledge scores, average number of professionals and caregivers trained, and professional trainee knowledge scores were evaluated by independent t-tests. The pre- and posttest scores between CPS and other SSIs for caregiver and professional training were compared using the nonparametric Mann–Whitney Wilcoxon U test. For professional trainings, pre- and posttest comparisons within groups were made using McNemar’s test for paired dichotomous variables (correct vs. incorrect). Statistical analyses were performed using SPSS for Windows, Version 23.0 (Armonk, NY, USA), with statistical significance set at alpha = 0.05. The ethical review and approval were waived by the University of Kansas School of Medicine Institutional Review Board, as this study involved secondary analysis of the deidentified data collected for program evaluation purposes.

## 3. Results

### 3.1. Fall 2019 SSI Certification Training Results

Training was attended by 43 participants, 27 (63%) of whom were employed by DCF CPS. The remaining attendees (*n* = 16; 37%) were employed in health care (e.g., hospitals), health departments, childcare licensing, and parenting and early childhood support programs. Prior to the training, CPS SSI trainees correctly answered an average of 7.5 questions out of 10 (SD = 1.2; range = 5–10) and other SSI trainees correctly answered an average of 8.6 questions out of 10 (SD = 1.0; range = 7–10) (*t =* 3.33, *p* = 0.002) (Table 1).

Following the training, the average score for CPS SSI trainees was 9.7 (SD = 0.2; range = 8–10) and other SSI trainees scored 9.9 (SD = 0.3; range = 9–10) (*t =* 1.42, *p* = 0.163). CPS SSI trainees successfully demonstrated improved knowledge on all topics of safe sleep (*t =* 8.53, *p* < 0.001), achieving high proficiency (>90%) on all questions except describing the diagnosis of SIDS (*n* = 23; 85% correct). Similarly, other SSI trainees demonstrated improved knowledge following the training (*t =* 4.44, *p* < 0.001); high proficiency was achieved on all questions. All trainees successfully passed the safe sleep crib demonstration.

Post-training, participants self-assessed safe sleep knowledge before and after the training on a scale from 1 (low) to 10 (high). CPS SSI trainees ranked their baseline knowledge at 5.3 (SD = 1.9) and their post-training knowledge at 9.1 (SD = 0.7), (*t =* 11.18, *p* < 0.001), whereas other SSI trainees ranked their baseline knowledge at 7.2 (SD = 1.8) and post-training knowledge at 9.1 (SD = 1.1), (*t =* 5.78, *p* < 0.001). The majority of CPS SSI trainees (*n* = 14; 52%) reported prior agreement with the AAP safe sleep recommendations, and the remainder (*n* = 13; 48%) reported positive shifts in agreement following the training. Other trainees also reported prior agreement with the AAP safe sleep recommendations (*n* = 10; 67%) or a positive shift in agreement following the training (*n* = 5; 33%). No difference in agreement with AAP safe sleep recommendations was observed between trainee groups (*p* = 0.356). All participants reported high satisfaction with the quality and content of the training (mean = 5 out of 5; SD = 0.2).

### 3.2. Post-Certification Dissemination of Safe Sleep Information by SSIs

#### 3.2.1. Parent and Caregiver Trainings

Knowledge was evaluated for 139 parent and caregivers from six Kansas counties; 53 (38%) trained by CPS SSIs (45 (85%) completed post-survey) and 86 (62%) by other SSIs (80 (93%) completed post-survey). Safe sleep parent and caregiver training sessions ranged in size from one to 30 trainees (median attendance per training = 2). CPS SSIs trained an average of 12.6 (SD = 11.0) parent and caregivers per training compared to 1.9 (SD = 1.9) by other SSIs (*t =* −2.18, *p* = 0.095). Caregivers who attended CPS-led trainings correctly identified safe sleep recommendations on 3.1 (SD = 1.0) out of four questions prior to the training and 3.8 questions (SD = 0.5) following training (*t =* −4.91, *p* < 0.001). Caregivers who attended trainings led by other SSIs correctly identified safe sleep recommendations on 2.9 questions (SD = 1.2) prior to the training and 3.8 (SD = 0.7) after training (*t =* −6.17, *p* < 0.001). There was no statistically significant difference between parent and caregivers trained by CPS SSIs or other SSIs (*p* = 0.754).

Examining the specific safe sleep recommendations assessed, following CPS-led training, parent and caregivers demonstrated a positive increase in intention to follow safe sleep practices related to only placing their baby on their backs to sleep (89% vs. 96%; *p* = 0.218), anticipated sleep locations and surfaces (77% vs. 98%; *p* = 0.003), and anticipated crib items (74% vs. 96%; *p* = 0.004). Most parents and caregivers (96%) planned to discuss safe sleep with others after attending the parent and caregiver training sessions (*p* = 0.001) (Table 2). Parents and caregivers trained by other SSIs also demonstrated a positive increase in intention to follow safe sleep practices related to anticipated sleep position (77% vs. 94%; *p* = 0.002), anticipated sleep locations and surfaces (78% vs. 95%; *p* = 0.001), anticipated crib items (66% vs. 94%; *p* < 0.001), and discussing safe sleep with others (64% vs. 98%; *p* < 0.001). No differences were observed between those trained by CPS and other SSIs.

#### 3.2.2. Professional Trainings

Knowledge was evaluated for 1216 professionals from 28 Kansas counties, with 1051 (86%) trained by CPS SSIs and 165 (14%) trained by other SSIs. Safe sleep professional trainings ranged in size from 1 to 59 trainees (median attendance per training = 13). One training was completed virtually due to restrictions to in-person meetings. CPS SSIs trained an average of 15.9 (SD = 10.7) professionals per training compared to other SSIs with 3.7 (SD = 6.4), (*t =* −8.51, *p* < 0.001). Prior to the training, professionals attending training sessions led by CPS SSIs correctly answered an average of 6.6 questions out of 10 (SD = 1.7; range = 0–10) and professionals trained by other SSIs correctly answered an average of 7.6 questions out of 10 (SD = 1.7; range = 0–10), (*t =* −6.80, *p* < 0.001) (Table 3).

Following the training sessions, the average score for CPS SSI-trained professionals was 9.0 (SD = 1.1; range = 3–10), a statistically significant improvement over the baseline (*t =* 44.75, *p* < 0.001). The average score for professionals trained by other SSIs was 9.2 (SD = 1.08; range = 6–10), also a statistically significant improvement over the baseline (*t =* 12.08, *p* < 0.001). A statistically significant difference was observed when comparing post-training scores between professionals trained by CPS SSIs and other SSIs (*t =* −2.54, *p* = 0.012). CPS SSIs were less likely to correctly answer questions related to the diagnosis of SIDS (81% vs. 89%; *p* = 0.012) and messaging strategies (87% vs. 92%; *p* = 0.047).

Professionals were asked to self-assess knowledge before and after the training on a scale from 1 (low) to 10 (high). Professionals trained by CPS SSIs stated that their knowledge rose from 6.2 (SD = 2.2) prior to the training to 9.0 (SD = 1.1) following the training (*t =* 47.18, *p* < 0.001), and professionals trained by other SSIs stated that their knowledge rose from 7.0 (SD = 2.1) to 9.3 (SD = 0.9), (*t =* 15.27, *p* < 0.001). There was a significant difference in self-assessment of knowledge between professionals trained by CPS and other SSIs (*t =* −3.71, *p* < 0.001). Professionals were also asked how the training affected their beliefs regarding the AAP recommendations. The majority of professionals trained by CPS SSIs reported prior agreement with the AAP safe sleep recommendations (*n* = 518; 51%), while 47% reported a positive shift in agreement (*n* = 482), 1% reported a negative shift in agreement (*n* = 9), and 1% reported no change (*n* = 12). In contrast, 66% of professionals trained by other SSIs reported prior agreement (*n* = 101), 33% reported a positive shift (*n* = 51), 0.5% reported a negative shift (*n* = 1), and 0.5% reported no change (*n* = 1). A statistically significant difference in agreement with AAP safe sleep recommendations was observed between professionals trained by CPS SSIs and those trained by other SSIs (*p* = 0.001).

## 4. Discussion

Family support and case-management services, such as those provided by DCF CPS, are potentially cost saving [17] and can significantly reduce the risk of child fatality [18]. Families who receive such services often view them positively and develop relationships with their support workers [19,20]. These collaborative relationships may create an optimal opportunity for safe sleep education to be provided in the home and with non-judgmental conversations [19,20], which has been recommended as the most effective way to engage caregivers in safe sleep education [21].

Most evaluations of CPS worker trainings fail to extend beyond the training itself [22]; however, SSIs were evaluated at all four levels recommended by Kirkpatrick (reaction, learning, behavior, and results). These data suggest that the KIDS Network SSI training of CPS staff was successful. Specifically, for level 1, reaction, we found that participants were satisfied with the quality and content of the training. For level 2, learning, we identified that CPS staff had significantly lower knowledge of the AAP recommendations prior to the training when compared to participants of other vocations (e.g., nurses and health department staff). However, CPS staff knowledge improved significantly by posttest (equivalent to other trainees), all successfully performed the safe sleep crib demonstration simulation, and all received SSI certification.

Level 3, behavior, was evaluated by tracking professional and parent/caregiver training sessions facilitated by the CPS SSIs. The CPS SSIs were required to disseminate safe sleep training to 10 professionals but trained over twice as many as expected and significantly more than other SSIs. Caregivers were also successfully trained by both groups of SSIs. Level 4, results, showed significant improvement in knowledge for both professionals and caregivers trained by the CPS SSIs. Professionals had significant improvements over baseline following training by CPS SSIs. However, professionals trained by other SSIs had significantly higher post-training knowledge scores and self-assessment scores compared to those trained by CPS SSIs. Following the SSI training, some CPS SSIs did not correctly identify the definition of SIDS on the post-assessment. While remediation was provided at the training, professionals trained by other SSIs showed significant improvement on this item, while those trained by CPS SSIs did not. Future CPS SSI-led training should focus more on the diagnosis of SIDS and strategies to communicate safe sleep messages, which was also higher for those trained by other SSIs. Differences in the outcomes for professionals trained by SSIs may also be due to the lower baseline knowledge of those trained by CPS SSIs or a reflection of smaller learning groups engaged by other SSIs. However, as the average scores for CPS and other SSI trainees on both measures were 9 or higher on a scale of 10, these results may not be meaningful.

The SSI training may have resulted in successful outcomes with CPS staff due to several factors. The SSI training not only provided training on educating multiple priority audiences (professionals and caregivers) in various formats (one-on-one, group) but also on approaching dissemination through a lens of cultural humility [23] and relationship building [19,20]. Designing a training program in this manner allows workers to take family characteristics into account and to adapt the training and education to meet individual needs while maintaining the fidelity of the program, which have been identified as important factors by CPS workers [24].

It is also important to recognize that a dose–response relationship has been found with safe sleep education, where more exposures increases the likelihood of parents following safe sleep recommendations, such as placing infants supine to sleep [6]. The strongest recommendations are those from health care professionals [25,26], but hearing consistent messages from CPS staff may increase the likelihood that caregivers utilize safe sleep practices. In addition, CPS staff have the opportunity to conduct sleep assessments in the parent or caregiver’s home, which is often limited in traditional safe sleep education strategies (e.g., parent education classes and Safe Sleep Community Baby Showers [15,16]). Further partnership should explore opportunities for in-home assessment of sleep environments and identification of barriers to safe sleep specifically for families engaged with CPS.

This study has several limitations. The project was conducted in a Midwestern state in the US, which may impact generalizability. It is also important to recognize that access to services may differ. For example, rural areas have been found to lack intensive family preservation services [27] and additional strategies for safe sleep education may be needed. Social desirability response bias may have impacted the self-report measures. Furthermore, the timing of the post-survey may have impacted the results as knowledge and skills may dissipate over time. This may be especially true for professionals and caregivers trained by SSIs as there was no post-training follow-up, whereas CPS and other SSIs were required to facilitate training that utilized the skills and knowledge from the training. Only parents and caregivers trained directly by SSIs were assessed, and thus, we are unable to measure the full extent to which safe sleep information was disseminated to additional families by the 1051 professionals trained by CPS SSIs. The behavioral result of those professionals and caregivers who received safe sleep training from the CPS SSIs was beyond the scope of this study. In addition, Kansas Child Death Review Board Reports will not be available for several years regarding data from families impacted by CPS SSIs, and even when the data are available, causation cannot be assumed.

Future studies should further partner with CPS to assess the behavioral impacts of the SSI program by extending data collection and follow-up to the families served, either directly by CPS SSIs or by CPS staff trained by SSIs. Priority questions include assessment of families’ understanding of the AAP safe sleep recommendations, actual infant sleep practices, and changes in sleep practices over time. In addition, replication in other locations or utilization of a control group would provide further evidence regarding the true impact of the intervention.

## 5. Conclusions

The innovative partnership with DCF CPS allowed for the provision of training to a group not historically targeted for safe sleep education. CPS SSIs showed significant increases in knowledge of the AAP safe sleep recommendations [2] following the certification training and equivalent knowledge to trainees from other organizations. Post-certification, CPS SSIs were able to increase the safe sleep knowledge of other CPS professionals in subsequent trainings, suggesting that the train-the-trainer model was successful, although future SSI trainings should address topics that CPS SSIs were less effective than other SSIs at disseminating to professionals. CPS SSIs were able to increase caregiver knowledge and to increase intentions to use safe sleep practices. Caregivers engaged with DCF have risk factors associated with sleep-related infant death, such as low socioeconomic status, and may be less likely to engage with other programs and resources. Furthermore, CPS staff engage regularly with clients, often in their own homes, providing a unique opportunity to address barriers, to provide support to strengthen families, to promote a nurturing environment for the child, and to assess behavior over time. 

## Figures and Tables

**Table 1:** Safe Sleep Instructor (SSI) trainees reporting only safe/correct responses for knowledge of American Academy of Pediatrics safe sleep recommendations.

	CPS SSI *n* = 27			Other SSI *n* = 16			
Concept	Pre-Survey	Post-Survey	within Group Change	Pre-Survey	Post-Survey	within Group Change	between Group Change Post-Survey
			*p*			*p*	*p*
SIDS Diagnosis	19 (70)	23 (85)	0.344	10 (63)	16 (100)	0.031 *	0.110
Surface and Location	8 (30)	26 (96)	<0.001 *	7 (44)	16 (100)	0.004 *	0.441
Position	21 (78)	27 (100)	0.031 *	15 (94)	16 (100)	1.000	1.000
Environment/Items	25 (93)	27 (100)	0.500	16 (100)	15 (94)	1.000	0.194
Environment/Tobacco	9 (33)	26 (96)	<0.001 *	12 (75)	16 (100)	0.125	0.441
Breastfeeding	27 (100)	27 (100)	n/a	15 (94)	16 (100)	1.000	1.000
Pacifier Use	26 (96)	26 (96)	1.000	16 (100)	16 (100)	n/a	0.441
Swaddling/Temperature	18 (67)	27 (100)	0.004 *	15 (94)	16 (100)	1.000	0.194
Twins and Higher Order Multiples	25 (93)	27 (100)	0.500	16 (100)	16 (100)	n/a	1.000
Shared Messaging	24 (89)	25 (93)	1.000	16 (100)	16 (100)	n/a	0.271

Note: the data are presented as *f (%)*. * *p*-value < 0.05 indicates a statistically significant difference between pre- and post-survey responses.

**Table 2:** Parents and caregivers reporting only safe/correct responses for intended safe sleep practices.

	CPS SSI-Led Training			Other SSI-Led Training			
Concept	Pre-Survey	Post-Survey	within Group Change	Pre-Survey	Post-Survey	within Group Change	between Group Change
	*n* = 53	*n* = 45	*p*	*n* = 86	*n* = 80	*p*	*p*
Sleep position: Back only	47 (89)	43 (96)	0.218	66 (77)	75 (94)	0.002 *	0.102
Anticipated sleep surfaces: Crib, bassinet, portable crib	41 (77)	44 (98)	0.003 *	67 (78)	76 (95)	0.001 *	0.893
Anticipated crib items: Only safe items (firm mattress, fitted sheet, wearable blanket)	39 (74)	43 (96)	0.004 *	57 (66)	75 (94)	<0.001 *	0.406
Discuss safe sleep with others	37 (70)	43 (96)	0.001 *	55 (64)	78 (98)	<0.001 *	0.764

Note: the data are presented as *f* (%). * *p*-value < 0.05 indicates a statistically significant difference between pre- and post-survey responses.

**Table 3:** Professionals reporting only safe/correct responses for knowledge of American Academy of Pediatrics safe sleep recommendations.

	CPS SSI-Led Training *n* = 1051			Other SSI-Led Training *n* = 165			
Concept	Pre-Survey	Post-Survey	within Group Change	Pre-Survey	Post-Survey	within Group Change	between Group Change Post-Survey
			*p*			*p*	*p*
SIDS Diagnosis	818 (78)	852 (81)	0.058	129 (78)	147 (89)	0.004 *	0.012 *
Surface and Location	162 (15)	703 (67)	<0.001 *	49 (30)	123 (75)	<0.001 *	0.050
Position	809 (77)	1039 (99)	<0.001 *	142 (86)	160 (97)	<0.001 *	0.055
Environment/Items	826 (79)	1031 (98)	<0.001 *	150 (91)	159 (96)	0.035 *	0.153
Environment/Tobacco	467 (44)	973 (93)	<0.001 *	112 (68)	151 (92)	<0.001 *	0.631
Breastfeeding	946 (90)	1021 (97)	<0.001 *	151 (92)	158 (96)	0.092	0.335
Pacifier Use	425 (40)	929 (88)	<0.001 *	111 (67)	154 (93)	<0.001 *	0.059
Swaddling/Temperature	727 (69)	1020 (97)	<0.001 *	131 (79)	163 (99)	<0.001 *	0.202
Twins and Higher Order Multiples	874 (83)	1001 (95)	<0.001 *	146 (89)	159 (96)	0.007 *	0.523
Shared Messaging	923 (88)	910 (87)	0.402	137 (83)	152 (92)	0.015 *	0.047 *

Note: the data are presented as *f (%)*. * *p*-value < 0.05 indicates a statistically significant difference between pre- and post-survey responses.

## Data Availability

Data are available upon request.
